# High-Throughput Mutation Profiling Identifies Frequent Somatic Mutations in Advanced Gastric Adenocarcinoma

**DOI:** 10.1371/journal.pone.0038892

**Published:** 2012-06-18

**Authors:** Jeeyun Lee, Paul van Hummelen, Christina Go, Emanuele Palescandolo, Jiryeon Jang, Ha Young Park, So Young Kang, Joon Oh Park, Won Ki Kang, Laura MacConaill, Kyoung-Mee Kim

**Affiliations:** 1 Division of Hematology-Oncology, Department of Medicine, Samsung Medical Center, Sungkyunkwan University School of Medicine, Seoul, Korea; 2 Center for Cancer Genome Discovery, Dana-Farber Cancer Institute and Harvard Medical School, Boston, Massachusetts, United States of America; 3 Department of Pathology, Samsung Medical Center, Sungkyunkwan University School of Medicine, Seoul, Korea; Kunming Institute of Zoology, Chinese Academy of Sciences, China

## Abstract

**Background:**

Gastric cancer is one of the leading cancer types in incidence and mortality, especially in Asia. In order to improve survival, identification of a catalogue of molecular alterations underlying gastric cancer is a critical step for developing and designing genome-directed therapies.

**Methodology/Principal Findings:**

The Center for Cancer Genome Discovery (CCGD) at the Dana-Farber Cancer Institute (DFCI) has adapted a high-throughput genotyping platform to determine the mutation status of a large panel of known cancer genes. The mutation detection platform, termed OncoMap v4, interrogates 474 “hotspot” mutations in 41 genes that are relevant for cancer. We performed OncoMap v4 in formalin-fixed paraffin-embedded (FFPE) tissue specimens from 237 gastric adenocarcinomas. Using OncoMap v4, we found that 34 (14.4%) of 237 gastric cancer patients harbored mutations. Among mutations we screened, PIK3CA mutations were the most frequent (5.1%) followed by p53 (4.6%), APC (2.5%), STK11 (2.1%), CTNNB1 (1.7%), and CDKN2A (0.8%). Six samples harbored concomitant somatic mutations. Mutations of CTNNB1 were significantly more frequent in EBV-associated gastric carcinoma (*P = *0.046). Our study led to the detection of potentially druggable mutations in gastric cancer which may guide novel therapies in subsets of gastric cancer patients.

**Conclusions/Significance:**

Using high throughput mutation screening platform, we identified that PIK3CA mutations were the most frequently observed target for gastric adenocarcinoma.

## Introduction

Gastric cancer is the fourth most common cause of cancer worldwide, and the second leading cause of cancer deaths worldwide. The American Cancer Society has estimated that 21,000 new cases of gastric cancer diagnosed in the United States in 2012, and that more than 10,000 Americans would die of gastric cancer during the year. [1] Despite recent efforts in multi-modality therapeutic approach for advanced gastric cancer, approximately half of patients who undergo curative surgical resection still develop loco-regional or distant metastases and die from the disease.[2]–[4].

Recently, an increased understanding of the biological driver events for solid tumors, coupled with advances in technologies used to detect somatic cancer alterations, has led to a rapid progress in personalized cancer medicine programs at several cancer centers. [5], [6] Gastric cancer is a heterogeneous disease with multiple environmental etiologies and aberrant pathways of carcinogenesis. Nevertheless, precise molecular subclassifications as well as a full landscape of oncogenic driver mutations have not been defined yet in gastric cancer. One of the known gastric cancer subtypes is EBV-GC which has distinct clinicopathologic features, and a relatively favorable prognosis. [7], [8] We have adapted a high-throughput genotyping platform to determine the mutation status of a large panel of known cancer oncogenes and tumor suppressor genes to identify the subsets of gastric cancer patients who may potentially benefit from targeted therapy (Table 1). [5] Procurement of fresh frozen tissue specimens is not always feasible in cancer patients; and thus, a high throughput platform which reliably genotypes cancer using paraffin embedded tissue samples is needed until more reliable rare cell sequencing becomes available in reality. The genotyping platform, termed OncoMap, employs mass spectrometric-based genotyping technology (Sequenom) to identify 474 oncogenic mutations in 41 commonly mutated genes (Table S1 for complete list) which are known to be oncogenic or targetable to drugs. We attempted to screen and segment gastric cancer patients according to genotypes in a large cohort of patients.

**Table 1:** List of genes screened for in OncoMap v4.

Gene	Number of mutations
ABL1	16
AKT1	1
AKT2	2
APC	14
BRAF	50
CDK4	1
CDKN2A	11
CSF1R	7
CTNNB1	33
EGFR	51
ERBB2	9
FGFR1	2
FGFR2	6
FGFR3	8
FLT3	9
GNA11	2
GNAQ	3
GNAS	3
HRAS	16
IDH1	3
IDH2	2
JAK2	1
JAK3	3
KIT	27
KRAS	24
MAP2K1	7
MET	6
MLH1	1
MYC	6
NPM1	3
NRAS	22
PDGFRA	20
PIK3CA	23
PIK3R1	15
PTEN	15
RB1	11
RET	14
SRC	1
STK11	12
TP53	7
VHL	7

## Results

In this study, we examined 237 gastric adenocarcinoma samples from which 47% (*n* = 111) of the specimens were diffuse type in Lauren’s classification and 24% (*n* = 58) were EBV positive. One hundred and seventeen patients were diagnosed at stage IV and 118 patients received palliative chemotherapy for gastric cancer. As listed in Table 1, we screened 474 oncogenic mutations in 41 commonly mutated genes such as ABL1, AKT, APC, BRAF, CDKN2A, CSF1R, CTNNB1, EGFR, ERBB2, FGFR, FLT3, FNA, HRAS, IDH, JAK, KIT, KRAS, MAP2K, MET, MLH1, MYC, NPM, NRAS, PDGFR, PIK3CA, PIK3R1, PTEN, RB1, RET, SRC, STK11, TP53, and VHL. In 6 carcinomas, two different somatic mutations were found concomitantly. In 27 stage IV metastatic patients without surgical specimens available for DNA extraction, endoscopic biopsies or biopsies from distant metastases were used for analysis. The clinicopathologic characteristics, EBV status, chemotherapy history and vital status at the last follow-up are provided in Table 2. Molecularly targeted agents are not widely used in gastric cancer for clinical practice and no patients were treated with molecularly targeted agents.

Overall, 34 of 237 patients (14.4%) harbored mutated oncogenes in their cancer tissue. Of the hotspot mutations we screened using OncoMap v4, the most commonly mutated oncogenes in gastric cancer were PIK3CA (*n = *12, 5.1%), p53 (*n = *11, 4.6%), APC (*n = *6, 2.5%), STK11 (*n = *5, 2.1%), CTNNB1 (*n = *4, 1.7%), and CDKN2A (*n = *2, 0.8%) (Table 3).

**Table 2:** Clinicopathologic characteristics.

		Total	EBV(−)	EBV(+)
		N = 237	N = 179	N = 58
Gender	F	69	59	10
	M	168	120	48
Age	mean	54.6	54.0	56.22
	median	55	55	57.5
Lauren’s classification	intestinal	62	44	18
	diffuse	111	93	18
	mixed	14	12	2
	indeterminate	23	3	20
Location	upper 1/3	23	16	7
	mid 1/3	123	79	44
	lower 1/3	76	73	3
	entire stomach	15	11	4
pT stage‡	invades lamina propria or submucosa	21	5	16
	invades muscularis propria or subserosa	94	57	37
	penetrates serosa	71	4	67
	invades adjacent structures	24	1	23
pN stage‡	no LN metastasis	45	7	38
	metastasis in 1–6 LNs	62	47	15
	metastasis in 7–15 LNs	41	38	3
	metastasis in >15 LNs	62	60	2
Distant metastasis†	no	170	114	56
	yes	67	65	2
AJCC stage	I	42	4	38
	II	40	14	26
	III	38	35	3
	IV	117	114	3
Chemotherapy	Adjuvant chemotherapy	69	51	18
	Palliative chemotherapy	118	114	4
Follow-up period (days)				
	range	35–3754	35–3754	234–3112
	Median	527	418	878

† clinical classification.

‡ inoperable cases.

*Abbreviations*, LN, lymph node; T, tumor, N, node.

**Table 3:** Frequency of mutations.

Gene	Amino acid	N	%	EBV(+)	EBV(−)	*P* value
PIK3CA	E542K	6	2.53	3	3	
	E545K	5	2.11	2	3	0.076
	E545G	1	0.42	1	0	
p53	R306*	4	1.69	0	4	
	R175H	3	1.27	1	2	
	R273C	3	1.27	0	3	0.303
	R248Q	1	0.42	0	1	
APC	Q1378*	5	2.11	2	3	
	T1556fs*3	1	0.42	0	1	0.636
STK11	P281L	5	2.11	1	4	–
CTNNB1	D32N	2	0.84	2	0	
	G34E	1	0.42	1	0	0.046
	S37F	1	0.42	0	1	
CDKN2A	R58*	2	0.84	1	1	0.245

N, total number of samples with mutation.

## Discussion

In total, we found that 34 (14.4%) of 237 gastric cancer patients harbored mutations when analyzed with mutations. PIK3CA mutations were the most frequent (5.1%) followed by p53 (4.6%), APC (2.5%), STK11 (2.1%), CTNNB1 (1.7%), and CDKN2A (0.8%) in gastric cancer. In a previous high-throughput profiling of gastric cancers using OncoMap v3, [5] percentage of samples with mutation was 38.2%, which is slightly higher than our incidences and the most frequent mutations were PIK3CA mutations (14.5%) followed by KRAS genes (7.7%) and PTEN (2.5%). Recent exome sequencing of gastric adenocarcinoma showed that samples with microsatellite instability (MSI) had an average of 31.61 somatic mutations per megabase of DNA, whereas the microsatellite-stable (MSS) gastric cancer samples had an average of 3.29, a difference of approximately tenfold. [9] Frequently mutated genes in exome sequencing data included TP53 (36% and 73%), PIK3CA (14% and 20%) and CTNNB1 (9% and 13.3%), [9], [10] which are much higher than we found. The limitations of OncoMap mass spectrometric genotyping approach such as finite number of specific point mutations that can be assayed and an inability to detect most tumor suppressor gene mutations outside “hotspot” regions would be a reasonable explanation.

According to our survey, PIK3CA, found in 5.1% (12 of 237) of cases, were the most frequent mutations and the prevalence is similar to previous reports. [11], [12] Few studies have reported on the frequency of PIK3CA mutations in the range of 4 to 25% of gastric cancers. [13] A relatively wide range of PIK3 mutation in previous reports (4 to 25%) may owe to the fact that PIK3CA mutation was studied in small cohort of archival tissue specimens (*n* = 12 to 94) or different stages of cancer analyzed among various studies. Extensive clinicopathologic correlative analyses or functional studies have not been actively investigated in this tumor type, [12] rendering limited implications of PIK3CA mutations in gastric cancer. There is an array of PIK3CA inhibitors and few of these drugs are being tested in early phase trials for gastric cancer. The treatment benefit from PIK3 inhibitor in PIK3CA mutated gastric cancer should be tested in clinical trials. Of note the frequency of PIK3CA mutations seemed higher in metastatic stage IV disease (N = 6/117, 5.1%) and stage II/III (n = 5/78, 6.4%) than stage IB (N = 1/42, 2.4%). Interestingly, three PIK3CA mutations were associated with concomitant mutations of APC or CTNNB1 genes.

The p53 gene product functions as a cellular gatekeeper and plays important roles in cell growth and division. [14] The mutational site of p53 in gastric cancer is wide and the reported incidence of p53 mutations ranges from 3.2 to 65% [15] and we observed in 4.6% of cases using a subset of known p53 mutation sites. The incidence of p53 mutation was significantly lower in EBV-GC (*n = *1) when compared with non-EBV-GCs (*n = *10), which is in line with previous reports that EBV infection might substitute mutations of p53 during the gastric tumorigenesis. [16].

APC mutations are rare in extracolonic cancers, including gastric carcinomas, with less than 10% of both differentiated and undifferentiated gastric carcinomas containing such mutations. [17] In our study, APC mutations were observed in 2.5% of cases, which is slightly lower than previous report. [18] STK11 mutations, found in 2.1% were observed in P281L within the kinase domain and all 5 patients with this mutation were stage IV with extensive lymph node metastases.

In 16 cases (6.75%), we identified MLH1 V384D variant in carcinoma samples. Further confirmation with corresponding normal tissue confirmed this change as a germ-line variant rather than *de novo* mutation in the cancer tissue. MLH1 V384D variant is found in 2.5% and 3% of Japanese, 5.2% of Korean, and 7.7% of Chinese, but is not observed in Western populations. [19], [20] The frequency of this variant matched in our series supporting a high sensitivity of the OncoMap platform. We did not detect mutations in KRAS, EGFR, PTEN, HRAS and BRAF in our study, suggesting these mutations are rare in gastric carcinomas. These observations are consistent with recent exome sequencing data showing no mutations of KRAS, EGFR, HRAS and BRAF in 37 fresh gastric carcinoma samples in Asian populations. [9].

Although the genomic era has rapidly arrived, whole genome sequencing or whole exome sequencing is not available yet in the clinic to comprehensively profile genomic aberrations. Furthermore, one of the major limitations at the moment is the limited availability of fresh frozen tissues, especially in metastatic cancer patients. Thus, we developed OncoMap platform which reliably interrogates “hotspot” mutations using paraffin-embedded specimens. Currently, OncoMap platform is the only high-throughput platform which was tested in >1,000 paraffin-embedded tissue specimens. [5], [21], [22] Until the routine use of whole genome or whole-exome sequencing is available at a reasonable cost and amenable to input nucleic acid from archival material, clinicians and pathologists need to utilize paraffin embedded tissues to interrogate multiple “hotspot” mutations. We screened for “hotspot” mutations in one of the largest gastric cancer cohort. The most frequent somatic mutation in gastric cancer was PIK3CA mutation which could be a potential therapeutic target in this population. Another important finding is that there were no “hotspot” mutations in the following genes which currently have drugs developed against: BRAF, EGFR, ERBB2, PDGFRA, PTEN, and RET. Hence, the drugs against these “hotspot” somatic mutations should be of low priority for development in gastric cancer.

With the advent of personalized genomic medicine, the utilization as well as validity of mutation profiling using materials from paraffin-embedded tissues widens the spectrum of patients who can be screened for druggable targets. Our study represents one of the largest studies which screened for the presence of somatic mutations in gastric cancer using paraffin-embedded tissues. Now we plan to screen for the presence of known targetable somatic mutations in all gastrointestinal cancer patients.

## Methods

### Specimens

For this study, we used 237 gastric cancer samples. All primary tumor samples were obtained from formalin-fixed paraffin-embedded tumor specimens based on 80% cutoff for tumor sample purity from a single institute. The quality of all DNA samples was ensured by independent quantification and quantitative PCR. The study was conducted after the approval from the Samsung Medical Center Institutional Review Board (SMC IRB). The primary tumor samples were all collected from Samsung Medical Center. The study was approved by the SMC IRB for informed consent waiver using archival tissues with retrospective clinical data. *Chi* square test was used and P values <0.05 were considered statistically significant in this study.

### Selections of Oncogene Mutations and Genotyping

Our current OncoMap v4 interrogates 474 mutations in 41 genes that are relevant for cancer (Table S1). OncoMap v4 is an expansion of Oncomap_v1 previously described by Macconaill et al, in 2009. [5] It interrogates frequently occurring somatic mutations in 41 known oncogenes and tumor suppressors, many of which are known to predict response or resistance to targeted therapies. The somatic mutations in Oncomap v4 were selected based on literature review and frequency of occurrence in tumor tissue as published in the ‘Catalogue of Somatic Mutations in Cancer’ [18] database. Genomic DNA was quantified using Quant-iT™ PicoGreen® dsDNA Assay Kit (Invitrogen) per manufacturer’s protocol. 250 ng DNA was used for a mutation analysis using Oncomap mass spectrometric genotyping based on the Sequenom MassARRAY® technology and (Sequenom, Inc, San Diego, CA) performed as previously described high-throughput oncogene mutation profiling in human cancer with some modifications. [5], [22] 100 ng of tumor-derived genomic DNA was subjected to whole genome amplification (WGA). Next, up to 18-multiplexed PCR was performed on tumor genomic DNA to amplify regions harboring loci of interest. After denaturation, PCR products were incubated with the probes that anneal immediately adjacent to the query nucleotide and mass spectrometric genotyping using iPLEX chemistries was performed (Sequenom Inc, San Diego, CA) extending the probes with 1 base in the presence of chain-terminating di-deoxynucleotides that generate allele-specific DNA products. The extension products were spotted onto a specially designed chip and analyzed by MALDI-TOF mass spectrometry to determine the mutation status based on the difference in mass of the mutant and wild type base.

Next, an automated mutation calling algorithm was performed to identify candidate mutations. Putative mutations were further filtered by a manual review and selected for validation using multi-base homogenous Mass-Extend (hME) chemistry with a maximum pooling of 6 assays on the remaining 150 ng DNA of each sample. Primers and probes used for hME validation were designed using the Sequenom MassARRAY® Assay Design 4.0 software, applying default multi-base extension parameters.

Only mutations found in iPLEX and confirmed by hME were considered as ‘validated mutations’. iPLEX candidate mutations that were not confirmed by hME were considered as invalidated and were not reported. Examples of all detected mutations were confirmed by standard, bidirectional Sanger sequencing.

## Supporting Information

Table S1 **List of Genes and Amino Acid changes screened for in Oncomap_v4.**
(DOC)
