# Supplementary material for: High-Throughput Mutation Profiling Identifies Frequent Somatic Mutations in Advanced Gastric Adenocarcinoma
Source: PLoS One. 2012 Jun 18;7(6):e38892. doi: 10.1371/journal.pone.0038892 (PMC3377730; doi:10.1371/journal.pone.0038892)
Supplement: Table S1 — List of Genes and Amino Acid changes screened for in Oncomap_v4. (DOC) [file pone.0038892.s001.doc]

Supplementary Table 1. List of Genes and Amino Acid changes screened for in Oncomap_v4.

| Gene and amino acid change | chromosome location and base change |
| --- | --- |
| ABL1_D276G | c.132737341A>G |
| ABL1_E255K | c.132728184G>A |
| ABL1_E255V | c.132728185A>T |
| ABL1_E355G | c.132738224A>G |
| ABL1_F317L | c.132738111C>G |
| ABL1_F359V | c.132738235T>G |
| ABL1_G250E | c.132728170G>A |
| ABL1_H396R | c.132740177A>G |
| ABL1_L248V | c.132728163C>G |
| ABL1_M244V | c.132728151A>G |
| ABL1_M351T | c.132738212T>C |
| ABL1_Q252H | c.132728177G>C |
| ABL1_Q252H | c.132728177G>T |
| ABL1_T315I | c.132738104C>T |
| ABL1_Y253F | c.132728179A>T |
| ABL1_Y253H | c.132728178T>C |
| AKT1_E17K | c.104317596C>T |
| AKT2_R371H | c.45433700C>T |
| AKT2_S302G | c.45434060T>C |
| APC_E1306* | c.112203106G>T |
| APC_E1309fs*4 | c.112203116_112203120del |
| APC_E1309fs*4 | c.112203117_112203121del |
| APC_E1309fs*6 | c.112203113insA |
| APC_E1379* | c.112203325G>T |
| APC_Q1338* | c.112203202C>T |
| APC_Q1367* | c.112203289C>T |
| APC_Q1378* | c.112203322C>T |
| APC_Q1429* | c.112203475C>T |
| APC_R1114* | c.112202530C>T |
| APC_R1450* | c.112203538C>T |
| APC_R876* | c.112201816C>T |
| APC_S1465fs*3 | c.112203583_112203584del |
| APC_T1556fs*3 | c.112203856insA |
| BRAF_D587A | c.140099644T>G |
| BRAF_D587E | c.140099643G>C |
| BRAF_D587E | c.140099643G>T |
| BRAF_D594E | c.140099622A>T |
| BRAF_D594G | c.140099623T>C |
| BRAF_D594V | c.140099623T>A |
| BRAF_E586K | c.140099648C>T |
| BRAF_F595L | c.140099619A>C |
| BRAF_F595S | c.140099620A>G |
| BRAF_G464E | c.140127886C>T |
| BRAF_G464R | c.140127887C>G |
| BRAF_G464V | c.140127886C>A |
| BRAF_G466A | c.140127880C>G |
| BRAF_G466E | c.140127880C>T |
| BRAF_G466R | c.140127881C>G |
| BRAF_G466V | c.140127880C>A |
| BRAF_G469A | c.140127871C>G |
| BRAF_G469E | c.140127871C>T |
| BRAF_G469R | c.140127872C>G |
| BRAF_G469R | c.140127872C>T |
| BRAF_G469S | c.140127870_140127872delinsGCT |
| BRAF_G469S | c.140127871_140127872delinsGA |
| BRAF_G469V | c.140127871C>A |
| BRAF_G596R | c.140099618C>G |
| BRAF_I592M | c.140099628T>C |
| BRAF_I592V | c.140099630T>C |
| BRAF_K601del | c.140099601_140099603del |
| BRAF_K601E | c.140099603T>C |
| BRAF_K601N | c.140099601T>A |
| BRAF_K601N | c.140099601T>G |
| BRAF_L597Q | c.140099614A>T |
| BRAF_L597R | c.140099614A>C |
| BRAF_L597S | c.140099614_140099615delinsGA |
| BRAF_L597V | c.140099615G>C |
| BRAF_N581S | c.140099662T>C |
| BRAF_R444W | c.140127947G>A |
| BRAF_S605F | c.140099590_140099591delinsAA |
| BRAF_S605N | c.140099590C>T |
| BRAF_T599_V600insTT | c.140099607delinsCGTAGTA |
| BRAF_T599I | c.140099608G>A |
| BRAF_V471F | c.140127866C>A |
| BRAF_V600A | c.140099605A>G |
| BRAF_V600D | c.140099604_140099605delinsAT |
| BRAF_V600E | c.140099604_140099605delinsTT |
| BRAF_V600E | c.140099605A>T |
| BRAF_V600K | c.140099605_140099606delinsTT |
| BRAF_V600L | c.140099606C>A |
| BRAF_V600L | c.140099606C>G |
| BRAF_V600M | c.140099606C>T |
| BRAF_V600R | c.140099605_140099606delinsCT |
| CDK4_R24H | c.56431697C>T |
| CDKN2A_D108Y | c.21961036C>A |
| CDKN2A_D84Y | c.21961108C>A |
| CDKN2A_E61* | c.21961177C>A |
| CDKN2A_E69* | c.21961153C>A |
| CDKN2A_E88* | c.21961096C>A |
| CDKN2A_H83Y | c.21961111G>A |
| CDKN2A_P114L | c.21961017G>A |
| CDKN2A_R58* | c.21961186G>A |
| CDKN2A_R80* | c.21961120G>A |
| CDKN2A_W110* | c.21961028C>T |
| CDKN2A_W110* | c.21961029C>T |
| CSF1R_L301* | c.149433237A>T |
| CSF1R_L301S | c.149433237A>G |
| CSF1R_Y969* | c.149413837A>C |
| CSF1R_Y969* | c.149413837A>T |
| CSF1R_Y969C | c.149413838T>C |
| CSF1R_Y969F | c.149413838T>A |
| CSF1R_Y969H | c.149413839A>G |
| CTNNB1_A13T | c.41241044G>A |
| CTNNB1_A21T | c.41241068G>A |
| CTNNB1_D32A | c.41241102A>C |
| CTNNB1_D32G | c.41241102A>G |
| CTNNB1_D32H | c.41241101G>C |
| CTNNB1_D32N | c.41241101G>A |
| CTNNB1_D32V | c.41241102A>T |
| CTNNB1_D32Y | c.41241101G>T |
| CTNNB1_G34E | c.41241108G>A |
| CTNNB1_G34R | c.41241107G>A |
| CTNNB1_G34R | c.41241107G>C |
| CTNNB1_G34V | c.41241108G>T |
| CTNNB1_S33C | c.41241105C>G |
| CTNNB1_S33F | c.41241105C>T |
| CTNNB1_S33Y | c.41241105C>A |
| CTNNB1_S37A | c.41241116T>G |
| CTNNB1_S37C | c.41241117C>G |
| CTNNB1_S37F | c.41241117C>T |
| CTNNB1_S37P | c.41241116T>C |
| CTNNB1_S37Y | c.41241117C>A |
| CTNNB1_S45A | c.41241140T>G |
| CTNNB1_S45C | c.41241141C>G |
| CTNNB1_S45F | c.41241141C>T |
| CTNNB1_S45P | c.41241140T>C |
| CTNNB1_S45Y | c.41241141C>A |
| CTNNB1_T41A | c.41241128A>G |
| CTNNB1_T41I | c.41241129C>T |
| CTNNB1_T41P | c.41241128A>C |
| CTNNB1_T41S | c.41241128A>T |
| CTNNB1_T41S | c.41241129C>G |
| CTNNB1_V22_G38del | c.41241071_41241121del |
| CTNNB1_V22A | c.41241072T>C |
| CTNNB1_W25_D32del | c.41241081_41241104del |
| EGFR_A750P | c.55209972G>C |
| EGFR_D761N | c.55210005G>A |
| EGFR_D761Y | c.55210005G>T |
| EGFR_D770_N771insN | c.55216506insAAC |
| EGFR_E734K | c.55209924G>A |
| EGFR_E746_A750>V | c.55209961_55209976delinsT |
| EGFR_E746_A750del | c.55209959_55209973del |
| EGFR_E746_A750del | c.55209960_55209974del |
| EGFR_E746_S752>A | c.55209961_55209978del |
| EGFR_E746_S752>V | c.55209961_55209979delinsT |
| EGFR_E746_T751>A | c.55209961_55209975del |
| EGFR_E746_T751del | c.55209960_55209977del |
| EGFR_E746K | c.55209960G>A |
| EGFR_G719A | c.55209202G>C |
| EGFR_G719C | c.55209201G>T |
| EGFR_G719D | c.55209202G>A |
| EGFR_G719S | c.55209201G>A |
| EGFR_G735S | c.55209927G>A |
| EGFR_G810D | c.55216625G>A |
| EGFR_G810S | c.55216624G>A |
| EGFR_H773R | c.55216514A>G |
| EGFR_I744_A750>VK | c.55209954_55209973delinsGTCAA |
| EGFR_K745R | c.55209958A>G |
| EGFR_L730F | c.55209912C>T |
| EGFR_L747_A750>P | c.55209962_55209972delinsGC |
| EGFR_L747_A750>P | c.55209963_55209972delinsC |
| EGFR_L747_E749del | c.55209963_55209971del |
| EGFR_L747_P753>Q | c.55209963_55209982delinsCA |
| EGFR_L747_P753>S | c.55209964_55209981del |
| EGFR_L747_R748>FP | c.55209965_55209968delinsCCCG |
| EGFR_L747_S752del | c.55209963_55209980del |
| EGFR_L747_T751>P | c.55209963_55209975delinsC |
| EGFR_L747_T751>S | c.55209964_55209975del |
| EGFR_L747_T751del | c.55209964_55209978del |
| EGFR_L858M | c.55227008C>A |
| EGFR_L858R | c.55227008_55227009delinsAG |
| EGFR_L858R | c.55227009_55227010delinsGT |
| EGFR_L858R | c.55227009T>G |
| EGFR_L861Q | c.55227018T>A |
| EGFR_N771_P772>SVDNR | c.55216508_55216511delinsGCGTGGACAACCG |
| EGFR_P733L | c.55209922C>T |
| EGFR_P753S | c.55209981C>T |
| EGFR_P772_H773insV | c.55216511insGGT |
| EGFR_S752_I759del | c.55209978_55210001del |
| EGFR_S752Y | c.55209979C>A |
| EGFR_S768I | c.55216499G>T |
| EGFR_T790M | c.55216565C>T |
| EGFR_V742A | c.55209949T>C |
| EGFR_V769_D770insASV | c.55216505_55216506delinsCCAGCGTGGAT |
| EGFR_V769_D770insASV | c.55216505insCCAGCGTGG |
| EGFR_W731* | c.55209917G>A |
| ERBB2_A775_G776insYVMA | c.35134521ins |
| ERBB2_A775_G776insYVMA | c.35134522ins |
| ERBB2_D769H | c.35133787G>C |
| ERBB2_G776S | c.35134523G>A |
| ERBB2_G776VC | c.35134522insTTT |
| ERBB2_L755P | c.35133745_35133746delinsCC |
| ERBB2_L755S | c.35133746T>C |
| ERBB2_M774_A775insAYVM | c.35134519ins |
| ERBB2_V777L | c.35134526G>T |
| FGFR1_P252T | c.38401366G>T |
| FGFR1_S125L | c.38405095G>A |
| FGFR2_C382R | c.123264764A>G |
| FGFR2_K310R | c.123269493T>C |
| FGFR2_N549K | c.123248024A>C |
| FGFR2_S252W | c.123269667G>C |
| FGFR2_S372C | c.123264793G>C |
| FGFR2_Y375C | c.123264784T>C |
| FGFR3_G370C | c.1775887G>T |
| FGFR3_G697C | c.1778129G>T |
| FGFR3_K650Q | c.1777687A>C |
| FGFR3_L794fs*23 | c.1778747delinsGA |
| FGFR3_R248C | c.1773362C>T |
| FGFR3_S249C | c.1773366C>G |
| FGFR3_S371C | c.1775890A>T |
| FGFR3_Y373C | c.1775897A>G |
| FLT3_D835del | c.27490640_27490642del |
| FLT3_D835E | c.27490640A>C |
| FLT3_D835E | c.27490640A>T |
| FLT3_D835H | c.27490642C>G |
| FLT3_D835V | c.27490641T>A |
| FLT3_D835Y | c.27490642C>A |
| FLT3_I836del | c.27490637_27490639del |
| FLT3_I836M | c.27490637G>C |
| FLT3_Y572C | c.27506341T>C |
| GNA11_Q209L | c.3069942A>T |
| GNA11_Q209P | c.3069942A>C |
| GNAQ_Q209L | c.79599308_79599309delinsAA |
| GNAQ_Q209L | c.79599308T>A |
| GNAQ_Q209P | c.79599308T>G |
| GNAS_Q227L | c.56917991A>T |
| GNAS_R201C | c.56917815C>T |
| GNAS_R201H | c.56917816G>A |
| HRAS_G12C | c.524289C>A |
| HRAS_G12D | c.524288C>T |
| HRAS_G12R | c.524289C>G |
| HRAS_G12V | c.524288C>A |
| HRAS_G13C | c.524286C>A |
| HRAS_G13R | c.524286C>G |
| HRAS_G13S | c.524286C>T |
| HRAS_G13V | c.524285C>A |
| HRAS_Q61H | c.523873C>A |
| HRAS_Q61H | c.523873C>G |
| HRAS_Q61K | c.523875G>T |
| HRAS_Q61L | c.523874T>A |
| HRAS_Q61P | c.523874T>G |
| HRAS_Q61R | c.523873_523874delinsAC |
| HRAS_Q61R | c.523873_523874delinsTC |
| HRAS_Q61R | c.523874T>C |
| IDH1_R132C | c.208821358G>A |
| IDH1_R132H | c.208821357C>T |
| IDH1_R132S | c.208821358G>T |
| IDH2_R140Q | c.88432938C>T |
| IDH2_R172K | c.88432842C>T |
| JAK2_V617F | c.5063770G>T |
| JAK3_A572V | c.17809009G>A |
| JAK3_P132T | c.17815215G>T |
| JAK3_V722I | c.17806696C>T |
| KIT_D52N | c.55256521G>A |
| KIT_D816H | c.55294077G>C |
| KIT_D816V | c.55294078A>T |
| KIT_D816Y | c.55294077G>T |
| KIT_E839K | c.55297451G>A |
| KIT_K550_K558del | c.55288339_55288365del |
| KIT_K558_E562del | c.55288363_55288377del |
| KIT_K558_V560del | c.55288363_55288371del |
| KIT_K642E | c.55288978A>G |
| KIT_L576P | c.55288418T>C |
| KIT_N822K | c.55294097T>A |
| KIT_N822K | c.55294097T>G |
| KIT_P585P | c.55288446C>T |
| KIT_T670I | c.55290276C>T |
| KIT_V559A | c.55288367T>C |
| KIT_V559D | c.55288367T>A |
| KIT_V559del | c.55288366_55288368del |
| KIT_V559G | c.55288367T>G |
| KIT_V559I | c.55288366G>A |
| KIT_V560D | c.55288370T>A |
| KIT_V560G | c.55288370T>G |
| KIT_V654A | c.55289015T>C |
| KIT_V825A | c.55294105T>C |
| KIT_W557G | c.55288360T>G |
| KIT_W557R | c.55288360T>A |
| KIT_W557R | c.55288360T>C |
| KIT_Y503_F504insAY | c.55286942insGCCTAT |
| KRAS_A146T | c.25269829C>T |
| KRAS_A59T | c.25271550C>T |
| KRAS_G12A | c.25289551C>G |
| KRAS_G12C | c.25289552C>A |
| KRAS_G12D | c.25289551C>T |
| KRAS_G12R | c.25289552C>G |
| KRAS_G12S | c.25289552C>T |
| KRAS_G12V | c.25289551C>A |
| KRAS_G13A | c.25289548C>G |
| KRAS_G13C | c.25289549C>A |
| KRAS_G13D | c.25289548C>T |
| KRAS_G13R | c.25289549C>G |
| KRAS_G13S | c.25289549C>T |
| KRAS_G13V | c.25289548C>A |
| KRAS_L19F | c.25289529C>A |
| KRAS_L19F | c.25289529C>G |
| KRAS_Q22K | c.25289522G>T |
| KRAS_Q61E | c.25271544G>C |
| KRAS_Q61H | c.25271542T>A |
| KRAS_Q61H | c.25271542T>G |
| KRAS_Q61K | c.25271544G>T |
| KRAS_Q61L | c.25271543T>A |
| KRAS_Q61P | c.25271543T>G |
| KRAS_Q61R | c.25271543T>C |
| MAP2K1_C121S | c.64516207T>A |
| MAP2K1_C121S | c.64516208G>C |
| MAP2K1_D67N | c.64514537G>A |
| MAP2K1_K57N | c.64514509G>C |
| MAP2K1_K57N | c.64514509G>T |
| MAP2K1_P124L | c.64516217C>T |
| MAP2K1_Q56P | c.64514505A>C |
| MET_H1112R | c.116204700A>G |
| MET_H1112Y | c.116204699C>T |
| MET_M1268T | c.116210710T>C |
| MET_T1010I | c.116199226C>T |
| MET_Y1248C | c.116210650A>G |
| MET_Y1248H | c.116210649T>C |
| MLH1_V384D | c.37042244T>A |
| MYC_A59V | c.128819821C>T |
| MYC_N101T | c.128819947A>C |
| MYC_P260A | c.128820423C>G |
| MYC_P57S | c.128819814C>T |
| MYC_S77F | c.128819875C>T |
| MYC_T73I | c.128819863_128819864delinsTT |
| NPM1_W288fs*12 | c.170770152insCATG |
| NPM1_W288fs*12 | c.170770152insCCTG |
| NPM1_W288fs*12 | c.170770152insTCTG |
| NRAS_A18T | c.115060253C>T |
| NRAS_G12A | c.115060270C>G |
| NRAS_G12C | c.115060271C>A |
| NRAS_G12D | c.115060270C>T |
| NRAS_G12R | c.115060271C>G |
| NRAS_G12S | c.115060271C>T |
| NRAS_G12V | c.115060270C>A |
| NRAS_G13A | c.115060267C>G |
| NRAS_G13C | c.115060268C>A |
| NRAS_G13D | c.115060267C>T |
| NRAS_G13R | c.115060268C>G |
| NRAS_G13S | c.115060268C>T |
| NRAS_G13V | c.115060267C>A |
| NRAS_Q61E | c.115058053G>C |
| NRAS_Q61H | c.115058051T>A |
| NRAS_Q61H | c.115058051T>G |
| NRAS_Q61K | c.115058053G>T |
| NRAS_Q61L | c.115058052_115058053delinsAA |
| NRAS_Q61L | c.115058052T>A |
| NRAS_Q61P | c.115058052T>G |
| NRAS_Q61R | c.115058052_115058053delinsCT |
| NRAS_Q61R | c.115058052T>C |
| PDGFRA_D842_D846>E | c.54846851_54846863delinsG |
| PDGFRA_D842_D846>G | c.54846850_54846863delinsGA |
| PDGFRA_D842_D846>N | c.54846849_54846861delinsA |
| PDGFRA_D842_H845>V | c.54846850_54846860delinsTT |
| PDGFRA_D842_H845del | c.54846849_54846860del |
| PDGFRA_D842_M844del | c.54846849_54846857del |
| PDGFRA_D842_S847>EA | c.54846851_54846866delinsGGCC |
| PDGFRA_D842I | c.54846849_54846850delinsAT |
| PDGFRA_D842V | c.54846850A>T |
| PDGFRA_D842Y | c.54846849_54846851delinsTAT |
| PDGFRA_D842Y | c.54846849G>T |
| PDGFRA_D846Y | c.54846861G>T |
| PDGFRA_H845_N848>P | c.54846859_54846868delinsC |
| PDGFRA_I843_D846del | c.54846852_54846863del |
| PDGFRA_I843_S847>T | c.54846853_54846864del |
| PDGFRA_R841_D842del | c.54846846_54846851del |
| PDGFRA_S566_E571>K | c.54835808_54835822del |
| PDGFRA_S566_E571>R | c.54835807_54835824delinsCGC |
| PDGFRA_S566_E571>R | c.54835809_54835823del |
| PDGFRA_V561D | c.54835793T>A |
| PIK3CA_C420R | c.180410674T>C |
| PIK3CA_E542K | c.180418776G>A |
| PIK3CA_E542Q | c.180418776G>C |
| PIK3CA_E545A | c.180418786A>C |
| PIK3CA_E545D | c.180418787G>C |
| PIK3CA_E545D | c.180418787G>T |
| PIK3CA_E545G | c.180418786A>G |
| PIK3CA_E545K | c.180418785G>A |
| PIK3CA_E545Q | c.180418785G>C |
| PIK3CA_G1049R | c.180434784G>C |
| PIK3CA_G1049S | c.180434784G>A |
| PIK3CA_H1047L | c.180434779A>T |
| PIK3CA_H1047R | c.180434779A>G |
| PIK3CA_H1047Y | c.180434778C>T |
| PIK3CA_H701P | c.180421554A>C |
| PIK3CA_M1043I | c.180434768G>A |
| PIK3CA_M1043I | c.180434768G>T |
| PIK3CA_N1068fs*4 | c.180434843insA |
| PIK3CA_N345K | c.180404247T>A |
| PIK3CA_P539R | c.180418768C>G |
| PIK3CA_Q546K | c.180418788C>A |
| PIK3CA_R88Q | c.180399570G>A |
| PIK3CA_Y1021C | c.180434701A>G |
| PIK3R1_D560_S565del | c.67626840_67626857del |
| PIK3R1_D560Y | c.67626841G>T |
| PIK3R1_E439del | c.67625310_67625312del |
| PIK3R1_G376R | c.67624894G>A |
| PIK3R1_G376R | c.67624894G>C |
| PIK3R1_K459_S460>N | c.67625370_67625372del |
| PIK3R1_N564D | c.67626853A>G |
| PIK3R1_N564K | c.67626855C>A |
| PIK3R1_N564K | c.67626855C>G |
| PIK3R1_R461* | c.67625374C>T |
| PIK3R1_R557_K561>Q | c.67626833_67626844del |
| PIK3R1_R574fs*27 | c.67626884_67626885del |
| PIK3R1_T576del | c.67626889_67626891del |
| PIK3R1_T576del | c.67626890_67626892del |
| PIK3R1_W583del | c.67627006_67627008del |
| PTEN_K267fs*9 | c.89707755del |
| PTEN_K6fs*4 | c.89614223_89614224del |
| PTEN_N323fs*2 | c.89710791insA |
| PTEN_N323fs*21 | c.89710797del |
| PTEN_P248fs*5 | c.89707696insA |
| PTEN_P248fs*5 | c.89707697insA |
| PTEN_R130* | c.89682884C>T |
| PTEN_R130fs*4 | c.89682885del |
| PTEN_R130G | c.89682884C>G |
| PTEN_R130Q | c.89682885G>A |
| PTEN_R173C | c.89701879C>T |
| PTEN_R173H | c.89701880G>A |
| PTEN_R233* | c.89707652C>T |
| PTEN_R335* | c.89710832C>T |
| PTEN_V317fs*3 | c.89710779_89710782del |
| RB1_C706F | c.47935878G>T |
| RB1_E137* | c.47817245G>T |
| RB1_E748* | c.47937165G>T |
| RB1_L199* | c.47821149T>A |
| RB1_L660fs*2 | c.47931844_47931847del |
| RB1_R320* | c.47839649C>T |
| RB1_R358* | c.47840686C>T |
| RB1_R455* | c.47851761C>T |
| RB1_R552* | c.47853539C>T |
| RB1_R556* | c.47853551C>T |
| RB1_R579* | c.47925169C>T |
| RET_A883F | c.42935573_42935575delinsTTT |
| RET_A883F | c.42935574_42935575delinsTT |
| RET_C634R | c.42929954T>C |
| RET_C634W | c.42929956C>G |
| RET_C634Y | c.42929955G>A |
| RET_D631_L633>E | c.42929947_42929952del |
| RET_D631G | c.42929946A>G |
| RET_D898_E901del | c.42935619_42935630del |
| RET_E632_A640>VRP | c.42929949_42929972delinsTGCGGC |
| RET_E632_L633>V | c.42929949_42929951del |
| RET_E632_L633del | c.42929948_42929953del |
| RET_E768D | c.42933846G>C |
| RET_F612_C620del | c.42929084_42929110del |
| RET_M918T | c.42937422T>C |
| SRC_Q531* | c.35465176C>T |
| STK11_D194N | c.1171487G>A |
| STK11_D194V | c.1171488A>T |
| STK11_E199* | c.1171502G>T |
| STK11_E199K | c.1171502G>A |
| STK11_E57fs*7 | c.1158077del |
| STK11_F264fs*22 | c.1172264_1172267del |
| STK11_G196V | c.1171494G>T |
| STK11_P281fs*6 | c.1172319del |
| STK11_P281L | c.1172319C>T |
| STK11_Q170* | c.1171415C>T |
| STK11_Q37* | c.1158021C>T |
| STK11_W332* | c.1174059G>A |
| TP53_G245S | c.7518273C>T |
| TP53_R175H | c.7519131C>T |
| TP53_R248Q | c.7518263C>T |
| TP53_R248W | c.7518264G>A |
| TP53_R273C | c.7517846G>A |
| TP53_R273H | c.7517845C>T |
| TP53_R306* | c.7517747G>A |
| VHL_F148fs*11 | c.10163301del |
| VHL_L158Q | c.10166480T>A |
| VHL_L85P | c.10158785T>C |
| VHL_L89H | c.10158797T>A |
| VHL_P81S | c.10158772C>T |
| VHL_R161* | c.10166488C>T |
| VHL_R167W | c.10166506C>T |
